# Modifier genes and Lynch syndrome: some considerations

**DOI:** 10.1186/s13053-022-00240-2

**Published:** 2022-09-10

**Authors:** Rodney J. Scott

**Affiliations:** Discipline of Medical Genetics, School of Biomedical Sciences and Pharmacy, University of Newcastle, Callaghan, NSW 2308 Australia

**Keywords:** Lynch syndrome, MSH2, MLH1, Modifier genes, Incidence, Survival

## Abstract

Lynch Syndrome (LS) is a highly variable entity with some patients presenting at very young ages with malignancy whereas others may never develop a malignancy yet carry an unequivocal genetic predisposition to disease. The most frequent LS malignancy remains colorectal cancer, a disease that is thought to involve genetic as well as environmental factors in its aetiology. Environmental insults are undeniably associated with cancer risk, especially those imparted by such activities as smoking and excessive alcohol consumption. Notwithstanding, in an inherited predisposition the expected exposures to an environmental insult are considered to be complex and require knowledge about the respective exposure and how it might interact with a genetic predisposition. Typically, smoking is one of the major confounders when considering environmental factors that can influence disease expression on a background of significant genetic risk. In addition to environmental triggers, the risk of developing a malignancy for people carrying an inherited predisposition to disease can be influenced by additional genetic factors that do not necessarily segregate with a disease predisposition allele. The purpose of this review is to examine the current state of modifier gene detection in people with a genetic predisposition to develop LS and present some data that supports the notion that modifier genes are gene specific thus explaining why some modifier gene studies have failed to identify associations when this is not taken into account.

## Introduction

LS is defined as a person who presents with a cancer related to a deficiency in DNA mismatch repair. Approximately 15% of all colorectal cancers are linked to a deficiency in DNA mismatch repair (MMR), suggesting that this syndrome is quite common [1]. Overall, somewhere between 2 and 3% of all colorectal cancer patients carry an inherited predisposition to LS as a result of pathogenic variants residing in one of four genes associated with DNA mismatch repair [2–6]. The focus of this short review is on patients who carry an inherited predisposition to LS by virtue of the fact that they are more likely to lose MMR capacity. The review does not address any genetic modifiers in people who do not carry a genetic predisposition to LS.

LS started out as the Cancer family G [7], evolved into Familial Cancer Syndrome [8], followed by Lynch I and Lynch II, then changed to Hereditary Non Polyposis Colorectal Cancer (HNPCC) [9] and is now named Lynch syndrome [10]. This inherited predisposition to epithelial malignancies is a result of germline pathogenic variants occurring in one of four genes involved in DNA mismatch repair, *MSH2, MSH6, MLH1* and *PMS2* [2–6]. Pathogenic variants in any one of the four genes result in an increased risk of developing colorectal and endometrial cancers as well as a number of other epithelial malignancies [11] but there are subtle differences in disease risk pertaining to each of the four genes. The genetic definition of a syndrome requires the person with the syndrome presents with a phenotype consistent with disease so in the context of LS, patients carry the predisposition to develop LS but until they do, they should not be classified as “having” LS.

With respect to cancer risk, *MSH2* and *MLH1* have similar risk profiles but are subtly distinct from one another whereas *MSH6* has a unique risk profile associated with colorectal cancer and endometrial cancer and *PMS2* appears, on prospective evidence, to be primarily associated with endometrial cancer and little else [12, 13]. Differences in survival are also observed in LS families that appear to be gene specific [13] thereby complicating any study aimed at assessing the influence of modifier genes on disease expression in LS. The dissimilar disease risk profiles associated with the four genes that are involved in LS points towards a diverse set of genetic modifiers that are not necessarily applicable to each gene. Intriguingly, the colorectal cancer polygenic risk score, which is relatively accurate in predicting cancer risk in the general population appears not to be of much value when applied to LS families [14].

This result suggests that the polygenic markers associated with colorectal cancer are different in LS compared to the general population. This implies that gene specific cancer risk (i.e. cancer risk specifically associated with *MSH2, MSH6, MLH1* or *PMS2*), is likely to be different depending on which gene results in the loss of DNA MMR activity. Given that there are four genes associated with one syndrome it may be some time before useful information could be forthcoming that reveals genetic modifiers that could be reliably used for risk assessment. This is not to say no in-roads into identifying modifier genes linked to disease risk in LS have occurred. This review will focus on more recent findings with respect to genetic modifiers of cancer risk in Lynch Syndrome.

## Problems associated with identifying modifier genes in Lynch syndrome

LS appears on the surface to be a disorder that is associated with all the hallmarks of being a readily assessable syndrome that should reveal, relatively easily, modifier genes that influence either the type of disease a patient is likely to present with or the age at which an individual manifests disease. The Prospective Lynch Syndrome Database (PLSD) has revealed subtle but important differences between patients who carry pathogenic variants in one of the four DNA mismatch repair genes known to be associated with LS [8]. It is now obvious that the design of a study aimed at revealing the actions of a modifier gene should be gene specific. Currently, the PLSD reveals that overall, cancer risk for individuals carrying pathogenic variants (PVs) in MSH2 or MLH1 are similar with an approximate risk of (any) cancer being just over 70% by 70 years of age. Patients carrying MSH6 PVs have a cancer risk of a little less than 55% by 70 years of age and PMS2 PV carriers are at much lower cancer risk at a little less than 20% at 70 years of age [12].

Tumour specific risks of disease reveal that colorectal cancer risk is greatest for MLH1 PV carriers (~ 45% by 70 years of age), followed by MSH2 (~ 35% at 70 years of age and MSH6 (20% by 70 years of age). When examining the second most frequently reported malignancy in LS, endometrial cancer, the gene specific differences in risk are quite different with MSH2 and MSH6 being associated with an approximate 50% risk at 70 years of age whereas MLH1 risk is ~ 34% risk by 70 years of age and for PMS2 the risk is ~ 24% by 70 years of age [12, 13]. This information suggests that the four mismatch repair genes are better described as intermediate risk genes whereas genes like APC or BRCA1 are considered high risk genes linked to colorectal cancer (APC PVs are associated with almost complete penetrance and BRCA1 PVs are very high at ~ 75% at 70 years of age) and breast cancer [15, 16], respectively.

This information is important when taking into consideration many studies that have been reported that grouped LS patients (irrespective of the disease gene) together when attempting to identify a modifier gene that impacted on the age of disease expression. Not taking into account gene specificity when conducting modifier gene studies is likely to result in a failure to identify any true modifying association [17].

To undertake an appropriate study that identifies a modifier gene, the population size is crucial since sufficient numbers of patients are required to unequivocally define a modifier gene. Followed by which subgroup of LS is of interest (e.g. MSH2 carriers, MLH1 carriers, the type of mutation (missense, nonsense etc.) female patients, any genotype/phenotype correlation etc.) and as precise a definition as possible with respect to what modification is being searched for (i.e. age of disease onset, the site of disease development, environmental triggers of disease). Finally, a modifier gene should have some relationship to one of the four mismatch repair genes associated with LS. At this point in time there are unlikely to be insufficient PMS2 pathogenic variant carriers to identify, with any degree of certainty, genetic modifiers of disease risk in this group.

A few studies have been undertaken that take into account some of the aspects listed above when considering the role of modifier genes in LS. Two of the more recent studies into modifier genes have focussed on single nucleotide polymorphisms (SNPs) that have been identified in genome wide association studies (GWAS) where telomerase reverse transcriptase (*TERT*) polymorphisms were associated with colorectal cancer outside of the context of LS [18]. Functional studies have also revealed that loss of MSH2 results in accelerated telomere shortening in normal human cell lines [19]. Telomere shortening results in telomere dysfunction and subsequent genomic instability which culminates in either tumour development or progression [20]. Other SNPs residing at various genomic loci were also identified that were considered as potential modifiers of colorectal cancer risk.

Seven polymorphisms (located according to genome build GRCh38p13) rs16892766 (intergenic, chromosome 8), rs3802842 (COLCA2, chromosome 11), rs4939827 (SMAD7, chromosome 18), rs4464148 (SMAD7, chromosome 18), rs6983267 (lncRNA CASC8, chromosome 8), rs4779584 (intergenic, chromosome 15) and rs10795668 (lncRNA LOC105376400, chromosome 10) connected with colorectal cancer risk were screened in a population of 1,119 participants derived from 424 families from The Netherlands, Poland and Australia who carried unequivocal pathogenic variants in either *MLH1* or *MSH2.* Unsurprisingly, when a combined analysis was undertaken (i.e. examining *MLH1* and *MSH2* pathogenic variant carriers together) no differences in the age of disease onset between carriers of any of the 7 SNPs were observed. When the study population was divided into *MLH1* and *MSH2* PV carriers, no difference in the age of disease diagnosis was observed for the *MSH2* PV carriers whereas for the *MLH1* PV carriers both the rs3802842 and rs16892766 polymorphisms were shown to be associated with a significant difference in the age of colorectal cancer diagnosis. Homozygote carriers of the variant rs3802842 allele were diagnosed with disease approximately 10 years earlier than their heterozygote or wildtype counterparts. Further stratification of the ages of disease onset was observed when carriers of rs3802842 and rs16892766 polymorphisms were analysed together. The data from this analysis revealed that there a proportionate increase in earlier disease development with an increasing number of risk alleles [21]. This result was specific to MLH1, however, the mechanisms behind this difference in the age of disease diagnosis remains to be fully explained.

Three polymorphisms in *TERT* (rs2075786, rs2736108 and rs7705526) were assessed with respect to their potential as modifiers in 1881 LS patients. In this study it was possible to examine MSH2, MSH6 and MLH1 separately to determine if any association was apparent. Since MSH2 is known to be involved in TERT promoter activity [22], a special interest in that relationship was focused on. A total of 705 participants were used for this study, all of whom carried a MSH2 pathogenic variant. 342 were diagnosed with cancer and 363 were cancer free. Similar numbers were screened who carried pathogenic variants in MLH1, which attests to the veracity of this study. Two SNPs, rs2075786 and rs2736108 appeared to have an effect on the age of colorectal cancer diagnosis whereas there was no effect observed in *MLH1* or *MSH6* PV carriers [23]. It was observed that heterozygote carriers of the rs2736108 SNP were at greater risk of cancer compared to their wildtype counterparts. The number of homozygous carriers of the rs2736108 SNP were minimal (46 in total) with only 27 cancer carriers and 19 cancer free carriers, which negated any meaningful interpretation. Carriers of the minor allele of rs2075786 were more likely to develop cancer at an earlier age compared to heterozygous or wildtype allele carriers [23].

The results from these two reports represent the largest groups of LS patients genotyped in the search for modifiers of disease expression in this syndrome. Even with large numbers of patients it was not possible to investigate gender differences in disease expression even when gene specific studies were undertaken. It was not possible to identify any genetic modifiers of disease in *MSH6* PV carriers in either of these reports and at this point in time there have been little, if any mention of modifier genes in this subgroup of patients. Notwithstanding, there were some hints within the data reported by Wiis et al. 2021, that modifier genes in *MSH6* PV carriers may be very different to those identified in *MLH1* and MSH2 PV carriers [23]. Unfortunately, knowledge about any modifiers genes influencing disease risk in *MSH6* PV carriers awaits the collection of larger cohorts of patients for any in-depth investigation.

## Conclusion

With increasing knowledge about gene specific differences associated with LS it is now necessary to re-define the role of modifier genes in LS such that statistically robust associations are identified that can be used to personalise prevention options for patients at risk of presenting with disease. Previous studies into modifier genes associated with LS, as tantalising as they may be, should be repeated in larger cohorts if we are interested in fully understanding LS. Finally, knowledge of modifier genes may provide specific insights into the molecular events that precede overt disease that may prove useful in the development of patient specific therapies.

## Data Availability

Not applicable.
